# Dr. Charles E. Annabel

**Published:** 1918-02

**Authors:** 


**Dr. Charles E. Annabel,** N. Y. University 1871, died at his home in Waverly, Dec. 20, from pneumonia, aged 66. He was a member of the staff of the Elmira Medical and Surgical Institute.

